# Disrupting pegRNA intramolecular complementarity via PBS and spacer sequence alterations can enhance prime editing efficiency

**DOI:** 10.1093/nar/gkag292

**Published:** 2026-04-21

**Authors:** Zsuzsanna Biczók, Sarah L Krausz, Dorottya A Simon, Eszter Tóth, Éva Varga, Tamás Annus, Flóra Huba, Máté Varga, Éva Bakos, Elfrieda Fodor, Ervin Welker

**Affiliations:** Institute of Molecular Life Sciences, HUN-REN Research Centre for Natural Sciences, Budapest H-1117, Hungary; School of Ph.D. Studies, Semmelweis University, Budapest H-1085, Hungary; Gene Design Kutató Fejlesztő Kft., Budapest H-6724, Hungary; Institute of Molecular Life Sciences, HUN-REN Research Centre for Natural Sciences, Budapest H-1117, Hungary; School of Ph.D. Studies, Semmelweis University, Budapest H-1085, Hungary; Institute of Molecular Life Sciences, HUN-REN Research Centre for Natural Sciences, Budapest H-1117, Hungary; School of Ph.D. Studies, Semmelweis University, Budapest H-1085, Hungary; Hun-Gén Technologies Kft, Budaörs H-2040, Hungary; Institute of Molecular Life Sciences, HUN-REN Research Centre for Natural Sciences, Budapest H-1117, Hungary; Institute of Molecular Life Sciences, HUN-REN Research Centre for Natural Sciences, Budapest H-1117, Hungary; Doctoral School of Multidisciplinary Medical Science, University of Szeged, Szeged H-6726, Hungary; Deparment of Genetics, ELTE Eötvös Loránd University, Budapest H-1117, Hungary; Deparment of Genetics, ELTE Eötvös Loránd University, Budapest H-1117, Hungary; Deparment of Genetics, ELTE Eötvös Loránd University, Budapest H-1117, Hungary; Institute of Molecular Life Sciences, HUN-REN Research Centre for Natural Sciences, Budapest H-1117, Hungary; Institute of Biochemistry, HUN-REN Biological Research Centre, Szeged H-6726, Hungary; Institute of Molecular Life Sciences, HUN-REN Research Centre for Natural Sciences, Budapest H-1117, Hungary; Institute of Biochemistry, HUN-REN Biological Research Centre, Szeged H-6726, Hungary

## Abstract

The length and sequence of the primer binding site (PBS) are critical for efficient prime editing, and its intramolecular complementarity with the prime editing guide RNA (pegRNA) spacer is a major drawback. We investigated the effects of these factors by literature analyses and by testing over 300 modified pegRNAs with weakened PBS-spacer interactions. It has been suggested that the effective PBS length for plasmid-delivered pegRNAs without end protection is considerably longer than what efficient priming requires due to exonuclease digestion of the PBS ends; however, analysing literature data of over 3000 pegRNAs revealed no significant shift in the optimal PBS length for epegRNAs compared to conventional pegRNAs. We also found improvement in editing efficiency with up to seven-fold when mismatches were introduced in the spacer or PBS sequence disrupting complementarity, although this effect is more pronounced with non-optimal PBS lengths. A combination of spacer mismatches and PBS deletions led to further editing improvements, even compared to the optimal PBS, although finding the best combination requires extensive optimization. Here, we achieved near-optimal editing efficiency in the majority of cases without the need for prior pegRNA optimization by using SPELL (Streamlined Prime Editing with fixed-Length PBS Leverage), a prime editing approach that employs a 17–20 nucleotide-long PBS with a single nucleotide deletion.

## Graphical Abstract

**Graphical Abstract:**
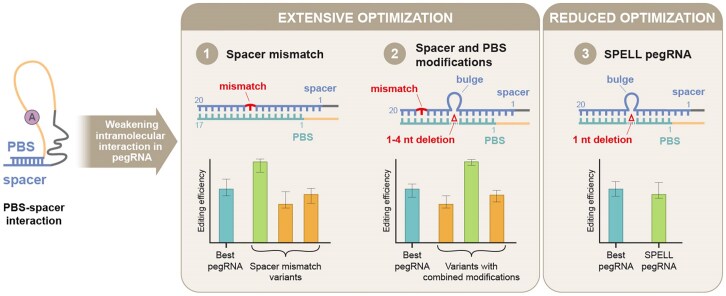

## Introduction

Prime editing (PE) is one of the newest generations of CRISPR-based genome modification tools, which does not require inflicting DNA double-strand breaks; therefore, it is expected to be potentially considerably safer than the conventional CRISPR nuclease-based approaches [1, 2]. PE can generate any type of substitutions, as well as small deletions and insertions through a complex multi-step process [1], [2]. Currently, intensive research efforts are put into increasing its efficiency, its effective editing window, and eliminating the need for its extensive, case-to-case based optimization [1, 3–15]. In a prime editor, a *Streptococcus pyogenes* Cas9 (SpCas9) nickase is fused to a reverse transcriptase, which extends the nicked 3′ DNA strand based on an RNA template designed by the user. Then, the extended DNA strand containing the desired modifications is integrated into the genome by the DNA repair machinery of the cell. In the prime editing gRNA (pegRNA), the 3′ end of the SpCas9 gRNA is extended by the RNA template, which contains (i) a right homology arm (RHA) for facilitating the integration of the modified DNA strand, (ii) the intended modified sequence and (iii) the primer binding site (PBS) which is complementary to the 3′ end of the nicked DNA strand [1]. This is the PE2 system, which has since been further developed in several ways to increase prime editing efficiency. PE3 was created by the introduction of an additional nick to the targeted DNA strand, which increases efficiency but also increases the indel background [1]. Efficiency can be further increased by the co-expression of the MLH1dn domain, which inhibits DNA mismatch repair, resulting in PE4 and PE5 from PE2 and PE3, respectively [3]. Optimizing the prime editor protein led to the development of PEmax [3], and engineered pegRNAs (epegRNAs) were also developed, containing a pseudoknot structure protecting the 3′ end of the pegRNA, therefore decreasing the formation of inhibitory truncated pegRNAs [5].

The length of the PBS is critical for the efficient priming of the reverse transcription, and it is thought to be limited by the adverse effect of its complementarity to the spacer part of the pegRNA. The optimal PBS length varies among targets, and despite a considerable effort to provide a guideline for its design, its determination requires extensive experimental optimization. Ponnienselvan *et al.* suggested that a PBS:non-target strand melting temperature (Tm) near 37°C is optimal in mammalian cells, Lin *et al.* suggested the use of 30°C Tm for prime editing in plants [11, 16]. Some increasingly accurate prediction software programs have also been developed [17–21]. Yu and Kim *et al.* have created pegRNA target libraries of impressive sizes and determined the PBS length at which they found the most efficient editing on average [17]. While these works may provide reasonable PBS lengths for PBS library design, they are less of a substitute for experimental determination of PBS length for individual targets.

A recent report challenged the commonly accepted perception regarding the optimal PBS length in prime editing experiments, particularly when using plasmid-based and lentiviral delivery of the pegRNA [16]. In their study, Ponnienselvan *et al.* compared the ideal PBS length for two edits, using either pegRNAs or epegRNAs [16]. Their findings revealed that pegRNAs with PBS lengths of 13 and 14 nucleotides resulted in more efficient editing than shorter (7 nucleotides) PBS lengths. However, when using epegRNAs, the significantly shorter PBS lengths of 7 nucleotides provided notably higher efficiency for both edits. This trend was consistent even when they employed ribonucleoprotein (RNP) delivery with synthetic, end-protected guide RNAs. The study proposed that the optimal PBS length observed in plasmid-based delivery may be longer due to the degradation of the 3′ end of the pegRNA in the cellular environment. When a longer PBS is used, the degradation process generates a distribution of PBS lengths, leading to a higher proportion of prime editing complexes containing the effective, shorter PBS. In contrast, the same pegRNA with the shorter PBS expressed directly from the plasmid may be degraded too much to be effective. However, when employing end-protected pegRNAs, where degradation is evaded, shorter PBS lengths with a PBS:target strand Tm near 37°C were found to be optimal [16]. This challenges the outcomes of the experiments investigating the effect of the length and nucleotide compositions of the PBS using pegRNAs without end protection.

**Figure 1.**
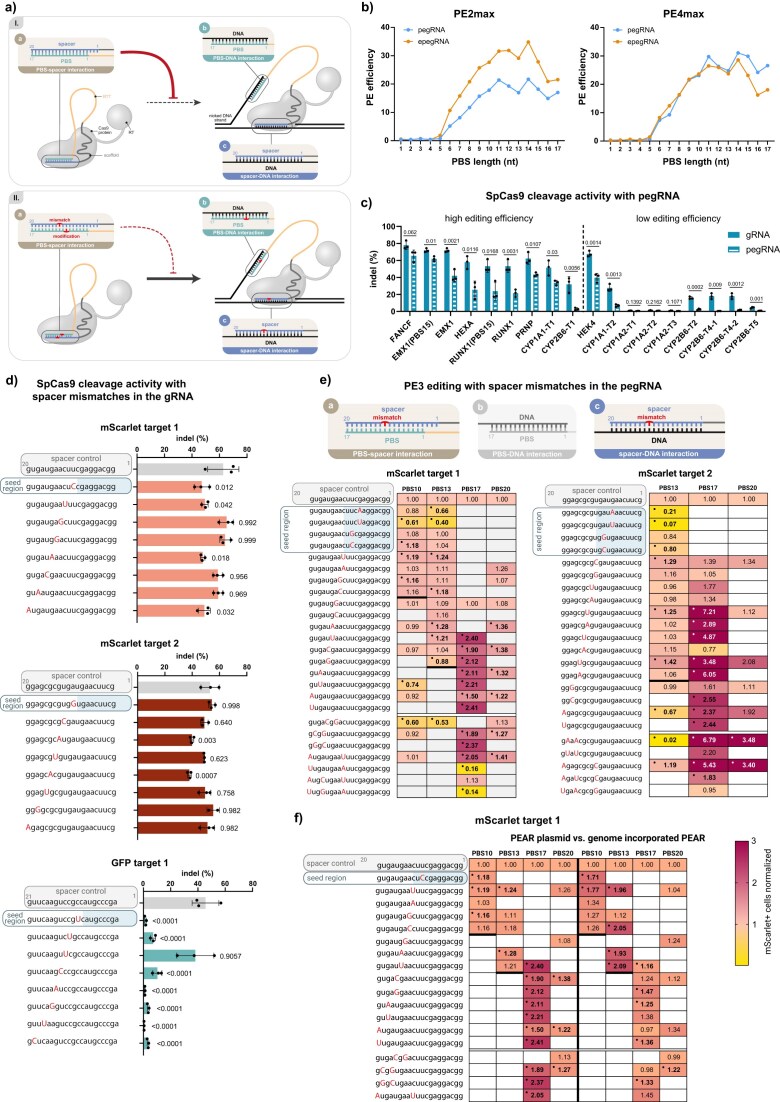
Mismatches in the pegRNA spacer can reduce self-complementarity and thus improve editing efficiency (**a**) Schematic figure showing self-complementarity between the pegRNA spacer and PBS, and the possible methods for its reduction. (**b**) Analysis of data derived from Yu and Kim *et al.* [17]. The mean PE efficiency of the pegRNAs and epegRNAs of varying PBS lengths, used either with PE2max or PE4max systems, was calculated from Library-Small and Library-epegRNA data. (**c**) Comparison of SpCas9 cleavage activities on endogenous targets in HEK293T cells for conventional sgRNAs and pegRNAs with 17 nt long PBS (for the *RUNX1-1, CYP1A1, CYP1A2*, and *CYP2B6* targets, epegRNAs were used). (**d**) Mismatch tolerance of SpCas9 on the three genome-incorporated PEAR targets. (**e, f**) The effect of spacer mismatches on PE3 efficiencies on plasmid-based PEAR targets in HEK293T cells (e) and in the genome-integrated PEAR system (right side of f). The PE3 efficiency values shown in the heatmaps were normalized to their corresponding no-mismatch-containing pegRNA control values. A significant deviation from the control is indicated by a dot in the upper left corner. Horizontal bold lines mark the end of complementarity between the spacer and PBS sequences. The three small schematic figures above the heat maps show the interacting sequences that may be affected by the modifications (those not affected are coloured grey). (c, d) Indel values were determined by using next-generation sequencing. The bars show the mean and the mean ± SD of *n* = 3 samples. The *P* values are shown above the columns, indicating deviations from the sgRNA (c) or from the no-mismatch control (d). d, e, f) On the vertical axis, the spacer sequences are presented in a 5′-3′ orientation, with mismatches in capital letters and highlighted in red. Differences between the sgRNAs and pegRNAs were tested for significance using unpaired two-tailed t-tests. The differences between the control and the samples containing a mismatching spacer were tested for significance using one-way ANOVA, Kruskal–Wallis test, or unpaired two-tailed t-tests. For all measured data and *P* values, see Supplementary Fig. S2 and Supplementary Data.

In this study, we found that the PBS length dependence of PE was not different between pegRNAs and epegRNAs and investigated ways to achieve more efficient prime editing by decreasing the PBS-spacer complementarity (Fig. 1a). We showed that mismatches introduced to the spacer or the PBS can increase PE efficiency. Deletions incorporated into long PBSs provided efficient editing comparable to that achieved with the optimal PBS lengths for the majority of the examined targets, allowing us to develop a universal approach coined SPELL (Streamlined Prime Editing with fixed-Length PBS Leverage), which may reduce the need for extensive optimization of the PBS.

## Materials and methods

### Materials

Restriction enzymes (BpiI (#ER1011) and BsmBI (#ER0451)), T4 ligase (#EL0012), Transcript Aid T7 High Yield Transcription Kit (#K0441), Dulbecco’s modified Eagle’s medium (DMEM) (#52100–047), Fetal Bovine Serum (#10 500 064), Turbofect Transfection Reagent (#R0532), Penicillin/Streptomycin (#15140–122), TrypLE Express Enzyme (#12 604 021), Geltrex LDEV-Free Reduced Growth Factor Basement Membrane Matrix (#A1413302) and Qubit dsDNA HS Assay kit (#Q32854) were purchased from Thermo Fischer Scientific. mTeSR1 media (#85 850) and Accutase (#07 920) were from STEMCELL Technologies. DNA oligonucleotides and the GenElute HP Plasmid Miniprep kit (# NA0160-1KT) used in plasmid purifications were acquired from Sigma-Aldrich. Q5 High-Fidelity DNA Polymerase (#M0491L), NEB Stable Competent *E. coli* (# C3040H) were from New England Biolabs Inc. NucleoSpin Gel and PCR Clean-up kit (#740609.250) was purchased from Macherey-Nagel. RNA Clean & Concentrator Kit (#R1018) was acquired from Zymo Research.

### Plasmid construction

The PEAR fluorescent reporter target plasmid with *GFP* (pDAS12125_PEAR-GFP, Addgene #177 178) and the pegRNA cloning plasmids (pDAS12222-U6-pegRNA-BFP, Addgene #177 181 and pDAS12069-U6-pegRNA-mCherry), were developed by Simon *et al.* [22]. The *mScarlet* fluorescent reporter plasmid (pAT9752-BEAR-mScarlet, Addgene #162 991) and the 2^nd^ nicking sgRNA coding plasmids (pAT9679-sgRNA-BFP (#162 988) and pAT9658-sgRNA-mCherry (#162 987)) were constructed by Tálas *et al.*(2021) [23].

For the construction of pegRNAs, a two-step cloning procedure was used: first, the spacer coding linkers were cloned into pDAS12222 plasmids between BpiI sites using three units of the BpiI enzyme, two units of T4 DNA ligase, 500 μM ATP, 1 × Green buffer, 50 ng vector, and 0.25 μM of each of the corresponding oligonucleotides. In the second cloning step, linkers containing the RTT-PBS were cloned between the BsmbI sites of the plasmids with the above-described method, but using a different buffer condition (1 mM DTT and 1 × Tango buffer). The sgRNA-expressing plasmids contain either an *mCherry* or a *TagBFP* expression cassette in order to monitor transfection efficiency.

For prime editing experiments, either the pCMV-PE2 plasmid (created by Anzalone *et al.* [1] and acquired from Addgene #132 775) or the same plasmid containing the PEmax modifications (R221K and N394K mutations in the SpCas9 nickase) was used as the PE and PEmax expressing plasmids. For non-prime editing experiments or for control samples, the X330-Flag-dSpCas9 (Addgene #92 113) plasmid was used as the dSpCas9-expressing plasmid, the pX330-Flag-wtSpCas9-H840A (Addgene #80 453) as the nSpCas9-expressing plasmid, and the pX330-Flag-wtSpCas9 (Addgene #92 353) [24] as the WT-SpCas9-expressing plasmid.

### Cell culturing and transfection

The HEK293T (CRL-3216) cells used were obtained from ATCC. The HuES9 human embryonic stem cell line was a gift from Dr. Douglas Melton, Harvard University. (HuES9 NIH Approval number: NIHhESC-09–0022 and Health Care Research Council, Human Reproduction Committee in Hungary, Approval number: 6681/2012-EHR.) The cell lines were authenticated by their respective suppliers, and they tested negative for mycoplasma during our experiments.

The HEK293T cells were cultured in DMEM supplemented with 10% heat-inactivated FBS, 100 units/mL penicillin, and 100 μg/mL streptomycin, at 37°C in a humidified atmosphere of 5% CO_2_. HuES9 cells were maintained on Geltrex-coated plates in mTeSR1 media at 37°C and 5% CO_2_. HuES cells were passaged every 4–5 days using the Accutase cell dissociation reagent and were placed in mTeSR1 supplemented with 10 µM ROCK inhibitor (Y-27632–2HCl, Selleckchem) for the first 24 hours.

In general, transfections of either of the cell types were performed in triplicate, and transfected cells were analyzed by flow cytometry three days post-transfection. Transfection efficiency was considered in both PEAR and genomic experiments. In PEAR experiments, only those mScarlet/GFP-positive cells were considered successfully edited that were also BFP/mCherry-positive (these were used to determine transfection efficiency and are found on pegRNA plasmids). For genomic experiments, samples were always analyzed by flow cytometry first, followed by genomic DNA purification. When evaluating sequencing data, samples were normalized for transfection efficiency.

#### Transfection of HEK293T cells

HEK293T cells were seeded on 48-well plates 1 day before transfection at a density of 3 × 10^4^ cells/well. For experiments using the PEAR system, the molar ratio of 3:5:1:1 of the plasmid components PE, pegRNS, 2^nd^ nicking RNA, and PEAR target plasmid, respectively, was used, as a total of 598 ng DNA, or 350 ng DNA per well. For the BEAR-*GFP* and BEAR-*mScarlet* cell lines (described by Tálas *et al.* [23], and Simon *et al.* [22]), a total of 598 ng of DNA was used at a 4:5:1 (PE:pegRNA:2^nd^ nicking RNA) molar ratio. In case of the genomic targets, the cells were transfected with a total of 350 ng of DNA per well at a 5:4:1 (PE2:pegRNA:2^nd^ nicking RNA or PEmax:pegRNA:2^nd^ nicking RNA for targets: *CDKL1, CTNNB1, CXCR4, IL2RB, HBG, COL7A1 T1, COL7A1 T2, CACNA1C T1*, and *CACNA1C T2*) molar ratio. For the wtSpCas9 experiments, 232 ng DNA was used per well at a 1:1.5 SpCas9:sgRNA molar ratio. Before transfection, the DNA was mixed with 1 μL Turbofect reagent diluted in 50 μL serum-free DMEM, and the mixture was incubated for 20–30 min at RT before adding it to the cells.

For the RNP experiments, cells were electroporated using the Lonza Amaxa 4D-Nucleofector. First, 50 pmol PEmax protein was mixed with 200 pmol *in vitro* transcribed pegRNA and incubated at room temperature for 10–15 minutes. Then the volume of the mix was supplemented with homemade electroporation buffer (described by Vriend *et al.* [25]) for a total volume of 10 µL. HEK293T cells were singularized by using TrypLE Express Enzyme, and 2 × 10^5^ cells were mixed with 10 µL homemade electroporation buffer. The RNP and cell mixtures were then mixed in a final volume of 20 µL and electroporated in a 16-well nucleocuvette strip using the CM-113 program. Transfected cells were plated on 48-well plates in 500 µL DMEM.

#### Transfection of HuES9 cells

HuES9 cells were transfected at ∼70% confluence with a total of 1050 ng DNA/cuvette at the same molar ratio as described previously for the genomic targets, in a final volume of 20 µL in a 16-well nucleocuvette strip. Two hours before electroporation, the media was changed to fresh mTeSR1 containing CEPT reagent (as described by Chen *et al.* [26]), then the cells were singularized using Accutase. 2 × 10^5^ cells were mixed with 20 µL homemade electroporation buffer (described by Vriend *et al.* [25]) containing the plasmid DNA, then they were electroporated using a Lonza Amaxa 4D-Nucleofector with the CA-137 program. Transfected cells were plated on Geltrex-coated 48-well plates in 500 µL CEPT-supplemented mTeSR1 and were incubated for 24 h, after which the media was changed to fresh.

### Flow cytometry

Flow cytometry analysis was carried out using an Attune NxT Acoustic Focusing Cytometer (Applied Biosystems by Life Technologies). As a rule, signals from a set target minimum of 20 000 viable single cells were acquired by gating based on the side and forward light-scatter parameters. BFP, GFP, mCherry, and mScarlet signals were detected using the 405 (for BFP), 488 (for GFP), and 561 nm (for mCherry and mScarlet) diode lasers for excitation, and the 440/50 (BFP), 530/30 (*GFP*), 620/15 (mCherry), and 585/16 nm (mScarlet) filters for emission. Attune Cytometric Software v.4.2 was used for data analysis. For the gating of live and GFP/BFP/mScarlet/mCherry positive cells, see Supplementary Fig. S1.

### Production and refolding of pegRNAs

The pegRNAs were synthesized by *in vitro* transcription. First, the DNA templates were constructed by PCR with the forward primer carrying a T7 promoter sequence. For the PCR, the Q5 High-Fidelity DNA Polymerase was used with the following program: 98°C, 40 s; 35 × (denaturation: 98 °C, 10 s; annealing: see Supplementary Table, 15 s; elongation: 72°C, 15 s; 72°C, 5 min. For the used primer sequences see Supplementary Table). The PCR products were purified using the NucleoSpin Gel and PCR Clean-up Kit. In vitro transcription was performed using the TranscriptAid T7 High Yield Transcription Kit at 37°C for 8 h and followed by the purification of the RNA by the RNA Clean & Concentrator^TM^-25 Kit.

The pegRNAs were refolded by heating to 98°C for 2 minutes and a slow cooling down, as described by Zhang *et al.* [27]

### PEmax protein expression and purification

The PEmax protein was overexpressed and purified using the bacterial expression plasmid PE-Max-pet21A (Addgene plasmid # 204 471) developed by the laboratory of Scot Wolfe [16] and by following the protocol of the Wolfe Lab [16, 28] with minor modifications and inclusion of a size exclusion chromatography step to the procedure. Briefly, *E. coli* Rosetta2(DE3)pLysS cells were transfected with the expression plasmid, and single colonies of transfected cells were picked to grow starter cultures in Luria-Bertani media at 37°C, up to an OD_600_ of about 2. For protein expression, rich growth media (16 g/l tryptone, 10 g/L yeast extract, 5 g/l NaCl, 755.8 mg/L NaH_2_PO_4_, 5.98 g/l Na_2_HPO_4_H_2_O, pH 7.5) of 3 × 1 L was inoculated by starter cultures to yield starting OD_600_s of 0.01, and the cells were grown at 37°C, 220 rpm, in an incubator-shaker. At the OD_600_ of approximately 0.6, the cultures were briefly chilled on ice (15–20 min), then shifted to 18°C, 220 rpm. At the OD_600_ of approximately 0.8, protein expression was induced by the addition of isopropylthiogalactoside to the media at a 0.7 mM final concentration. The induced cultures were grown for 18 h at 18°C. Cells were harvested by centrifugation (6000 g, 20 min, 4°C) and were resuspended in Ni-NTA buffer (20 mM Tris, 1 M NaCl, 20 mM imidazole, 1 mM TCEP, pH 7.5) supplemented by EDTA-free complete protease inhibitor cocktail tablets (Roche), lysozyme (∼ 0.1 mg/ml) and benzonase nuclease (∼5 units/mL), in order to proceed for cell lysis using repeated 30 s pulse-sonication on ice. The cell lysate was cleared by centrifugation (50 000 g, 50 min, 4°C), and then it was applied to Ni-NTA resin preequilibrated in Ni-NTA buffer. The lysate-loaded resin was incubated on ice for 1 h in 50 mL conical tubes, while gently shaking on a shaker, to capture the His-tagged proteins. The resin with bound proteins was poured onto empty chromatography columns, was washed by 5xCV (column volume) Ni-NTA wash buffer (20 mM Tris, 500 mM NaCl, 20 mM imidazole, 1 mM TCEP, pH 7.5) and the proteins were eluted from the resin in one step by 250 mM imidazole-elution buffer (20 mM Tris, 500 mM NaCl, 250 mM imidazole, 1 mM TCEP, 10% w/v glycerol, pH 7.5). The protein eluate was further purified by cation exchange chromatography using an UNOsphere-S column (Bio-Rad), and a gradient elution with buffers A (20 mM HEPES, 100 mM NaCl, 1 mM TCEP, 10% w/v glycerol, pH 7.5) and B (20 mM HEPES, 1 M NaCl, 1 mM TCEP, 10% w/v glycerol, pH 7.5), while collecting fractions of 1 ml. The fractions containing the PEmax protein confirmed by SDS-PAGE were combined, they were filtered through a 0.22 µm filter, and concentrated by centrifugation using 100 kDa cut-off concentrator tubes (100K MWCO, Pierce). The concentrated protein sample was then subjected to size exclusion chromatography using an ENrich-SEC650 high-resolution column (Bio-Rad) and SEC-buffer (20 mM HEPES, 500 mM NaCl, 1 mM TCEP, 10% w/v glycerol, pH 7.5) and collecting 0.75 mL fractions. The fractions containing the primary PEmax protein peak were combined, filtered through a 0.22 µm filter in a sterile hood, and concentrated by centrifugation (in 100K MWCO concentrator tubes) to at least 50 µM protein. The concentrated protein solution was distributed into small aliquots under sterile conditions and was flash-frozen in liquid nitrogen and placed at −80°C until use.

### Genomic DNA purification and genomic PCR

After flow cytometry, genomic DNA was extracted using the Puregene DNA Purification protocol (Gentra Systems Inc). Amplicons for next-generation sequencing were generated from the genomic DNA samples using two rounds of PCR to attach the Illumina handles. (The 1st step PCR primers used to amplify target genomic sequences are listed in the Supplementary Table.) PCR was done in an S1000 Thermal Cycler (Bio-Rad) or PCRmax Alpha AC2 Thermal Cycler using Q5 high-fidelity polymerase supplemented with Q5 buffer, and 150 ng of genomic DNA in a total volume of 25 μL. The thermal cycling profile of the PCR was: 98°C, 30 s; 35 × (denaturation: 98°C, 20 s; annealing: see Supplementary Table, 20 s; elongation: 72°C, 20 s; 72°C, 5 min. i5 and i7 Illumina adapters were added in a second PCR reaction using Q5 high-fidelity polymerase with supplied Q5 buffer and 1 µL of the first step PCR product in a total volume of 25 µL. The thermal cycling profile of the PCR was: 98°C, 30 s; 35 × (98°C, 20 s; 67°C, 30 s; 72°C, 20 s); 72°C, 2 min. Amplicons were purified by agarose gel electrophoresis. Samples were quantified with the Qubit dsDNA HS Assay kit and pooled.

### Next-generation sequencing

Sequencing on an Illumina NextSeq instrument was performed by Delta Bio 2000 Ltd. Reads were aligned to the reference sequence using BBMap. The aligned reads were considered as the total reads for each sample.

Indels at the pegRNA and 2^nd^ nicking sgRNA target sites were computationally identified from the aligned reads. Indels were searched for at ±2 bp around the nick/cut sites. For each sample, indel frequency was determined as (number of reads with an indel)/(number of total reads). The frequency of single-substitution mutations without indels generated by prime editing was determined as the percentage of (sequencing reads with the intended modification without indels)/(number of total reads). Conversely, the frequency of intended insertions or deletions generated by prime editing was determined as the percentage of (all sequencing reads with the intended modification)/(number of total reads). For these samples, the indel background was calculated from reads containing different types of indels than the intended edit. In both cases, reads with the intended modifications were identified by searching for a sequence stretch containing the desired edit flanked by 5–5 matching nucleotides. When calculating the edit or indel percentage for each sample, the respective background edit or indel percentage derived from untransfected cells was subtracted.

The following programs were used to analyze the NGS data: BBMap 38.08, Samtools 1.8, BioPython 1.71, and PySam 0.13.

For each CYP gene, gene-specific primers were used, and gene-specific reads were identified based on sequence differences between the two genes. Reads derived from non-gene-specific primer-annealing and from mixed PCR products due to template switching were excluded by exploiting two gene-specific motifs located at different positions of the amplicon.

### Analysing data from Yu and Kim *et al.*

We analyzed datasets 3, 4, 7, and 8 from the Supplementary Table S2 in the publication of Yu and Kim *et al.* [17] to compare the average PE efficiencies of pegRNAs grouped according to their PBS length between Library-Small and Library-epegRNA, when used with PE2max and PE4max systems. We included editing data in the analyses only from editing via the conventional ‘NGG’ PAM. In the case of some pegRNAs, we identified more and different editing data for a single pegRNA in the data files; therefore, we excluded these pegRNAs from our analysis.

### Statistics

Statistical significance was assessed by a two-tailed unpaired or paired t-test for comparing two groups. For comparing more than two groups, homogeneity of variances was tested by the Brown–Forsythe test, and normality of residuals was tested by the D’Agostino–Pearson omnibus (K2) test. For data sets with normal distributions, statistical significance was assessed using one-way ANOVA with Tukey’s post-hoc test (when comparing each group to every other group) or with Dunnett’s test (when comparing each group to a control group). In cases where data did not pass normality but fulfilled the assumptions of the Box-Cox transformation, the transformed data were analyzed as above. If not, the Kruskal–Wallis test with Dunn’s test was applied. Statistical tests were performed using GraphPad Prism 9.1.2.

## Results and discussion

Ponnienselvan *et al.* [16] examining two edits stated that the optimal PBS length for end-protected pegRNAs is relatively short—approximately 7 nucleotides—as opposed to the 12–14 nucleotide PBS lengths that typically perform best when pegRNAs are delivered by plasmid or viral vectors without end protection. However, a 7 nt long PBS is complementary only to spacer positions 3–10, therefore, if the functional PBS was restricted to these 7 nucleotides, then mismatches placed beyond spacer position 10 would have no effect on PBS:spacer annealing, whereas mismatches within positions 3–10 would be expected to reduce SpCas9 nicking activity more than how much they would reduce spacer:PBS inhibition. Therefore, this conclusion of Ponnienselvan *et al.*, if broadly applicable, would imply that our approach of disrupting PBS:spacer annealing by introducing mismatches or deletions would not work.

In order to find out whether the findings of Ponnienselvan *et al.* apply to all pegRNAs, we analyzed data from Yu and Kim *et al.* [17], who published efficiency measurements for ∼3000 pegRNAs and epegRNAs using both PE2max and PE4max editors [17]. Whilst determining the dependence of prime editing efficiency on PBS length, we found no clear difference in the PBS length that supports maximal average editing efficiency for pegRNAs with or without end protection. For both pegRNAs and epegRNAs, the highest average activities were observed with PBS lengths of 11 and 14 nucleotides, and there was no apparent shift of the optimal PBS length towards 7–8 nucleotides for end-protected pegRNAs (Fig. 1b). Thus, we concluded that plasmid-based delivery of pegRNAs without end protection provides a relevant and appropriate experimental context for our study.

### Spacer mismatches can increase prime editing efficiency

The efficiency of prime editing is decreased with long PBSs [17, 18]. We found that the cleavage activity of pegRNAs with 17-nucleotide-long PBSs is significantly lower or even completely diminished compared to that with their single guide RNA (sgRNA) counterparts. These data also show low prime editing efficiency with 17 nt PBSs when the cleavage activity of the corresponding sgRNAs was also low. (Fig. 1c). The complementarity of the PBS and the spacer, which may contribute to decreased efficiencies with 17 nt PBS, can be reduced by introducing mismatches to the spacer sequences. The mismatch tolerance of the SpCas9 is target- and sgRNA-dependent; it tolerates PAM-distal mismatches well for most targets, but there are a few exceptions where cleavage is inhibited completely [29–31]. We analyzed its mismatch tolerance, aiming at three PEAR plasmid targets integrated into the genome of HEK293T cells [23], the same targets we later examined using the plasmid-based PEAR system. The PEAR system [22], used in either plasmid-based or genome-integrated form, offers easy fluorescence readout to report on PE efficiency by the correction of an inactive splice site of a split *mScarlet* or *EGFP* gene separated by an intron sequence. (Supplementary Fig. S2a). To assess its mismatch tolerance on the selected targets, SpCas9 nuclease was used with mismatched sgRNAs, and the results were obtained using new generation sequencing (NGS) (Fig. 1d). In the case of the two *mScarlet* targets, SpCas9 showed cleavage activities with mismatching spacers above 80% of that of the perfectly matching spacer, while on the *GFP* target, it was substantially less tolerant for mismatches (Fig. 1d). This latter effect may be related to the use of 21G-sgRNAs, as discussed later. Exploring the observation, reported in the literature [32] and supported by two of the targets above, that SpCas9 generally tolerates one mismatch well at PAM distal positions between the target and the spacer of the sgRNA, we tested if the efficiency of PE can be increased by decreasing PBS-spacer complementarity (Fig. 1e). First, we used two *mScarlet* targets shifted by 7 nucleotides (Supplementary Fig. S2a) to introduce an AC to GT substitution [22] and designed 18 single-nucleotide and several two- or three-nucleotide mismatches placed primarily into the PAM-distal region of the two spacers (Fig. 1e; Supplementary Fig. S2b, c). We investigated the effect of spacer mismatches on PE3 activity using PBSs of different lengths (10, 13, 17, and 20 nucleotides, the latter discussed later). To define the limits of the effect, we also introduced mismatches at positions 8 and 10 of the spacer, as well as into the portion of the spacer outside the PBS-complementary region.

Most single mismatches in the PAM-distal (beyond position 10) PBS-complementary region increased PE efficiency with up to 7.2-fold (Fig 1e). Out of these 46 mismatching pegRNAs, one decreased the editing efficiency, while 24 significantly increased it. As expected, beyond the PBS-complementary region, no mismatches increased the efficiency of prime editing, while 2 mismatches out of the 7 significantly decreased PE efficiency. At the PAM-proximal positions 8 and 10, however, 7 out of the 12 mismatches significantly decreased PE efficiency, suggesting that PAM-proximal mismatches between the target DNA and the spacer caused a greater inhibition to the process than how much the reduced complementarity between the spacer and the PBS benefited it. (Fig. 1e, Supplementary Fig. S2b, c)

These results confirm that the efficiency of prime editing can be increased by introducing single mismatches to the PBS-complementary region of the spacer of the pegRNAs. Apparently, weakening the spacer-PBS interactions at PAM-distal positions can compensate for, or even outweigh, the negative effect of disrupting spacer–target pairing in the case of most mismatches placed within the PBS-complementary region of the spacer in the targets tested above.

We also examined the third PEAR target on which SpCas9 showed little mismatch tolerance (Fig 1d). As expected, none of the mismatches increased editing efficiency, in accordance with the above interpretation of the impact of the mismatch tolerances (Supplementary Fig. S3a, b)

We hypothesize that SpCas9 likely opens the two DNA strands at the target sequence by a few nucleotides longer than the 20-nucleotide-long segment complementary to the spacer. Thus, extending the length of the PBS to more than 17 nucleotides may increase the length of the PBS:non-target DNA strand duplex, which could potentially increase the efficiency of the reverse transcription initiation at certain targets, without elongating the inhibitory spacer-PBS complementarity. Increasing the PBS length to 20 nucleotides enhanced PE efficiency for one of the two targets tested (Supplementary Fig. S2d). However, the corresponding mismatches did not increase editing efficiency more with PBS20 than with PBS17. (Fig. 1e, Supplementary Fig. S2b, c)

We also tested the effect of introducing multiple mismatches simultaneously into the spacer. When all mismatches were in the PBS-complementary region, 10 out of 17 pegRNAs resulted in a significant increase in editing efficiency, while in the case of 3 pegRNAs, a significant decrease was observed (Fig. 1e, Supplementary Fig. S2b, c). This indicates that although multiple mismatches may have a higher beneficial impact on PE efficiency, they may cause a decrease in efficiency more often than single mismatches do.

To confirm that disrupting intramolecular self-complementarity can enhance editing efficiencies not only in the plasmid-based PEAR system but also in a genomic context, we tested the effect of spacer mismatches using 26 mismatching pegRNAs on *mScarlet* target 1 integrated into the genome (Fig. 1f, Supplementary Fig. S2e). Consistent with the plasmid-based assays, none of the spacer mismatches were detrimental to editing efficiency, with improvements of up to two-fold, even with the shorter (10 and 13 nt) PBS lengths.

### Mismatches in the PBS can also increase prime editing efficiency

Evidently, reducing the complementarity between the spacer and the PBS can increase the efficiency of PE. However, we hypothesized that introducing mismatches into the PBS instead of the spacer might be more beneficial for certain targets where SpCas9 has a low tolerance for spacer mismatches.

We considered three effects of mismatches regarding their impact on editing efficiency. (i) Mismatches can reduce the stability and the formation of the PBS:non-target strand heteroduplex, which can negatively influence the initiation of reverse transcription, and thus, the efficiency of PE. (ii) Reverse transcriptase (RT) forms non-sequence-specific interactions with the phosphate groups and sugar backbone of the heteroduplex, specifically with the 3′ nucleotides of the DNA primer and the 5′ nucleotides of the RNA. Thus, mismatches in this region can reduce the binding of RT to the heteroduplex and ultimately decrease the efficiency of PE. (iii) Mismatches reduce the complementarity between the spacer and PBS, which can lead to a reduction of inhibitory effects that, in turn, may increase the efficiency of prime editing. This effect is likely stronger if the mismatch occurs in the middle of the PBS rather than at its ends [33].

**Figure 2.**
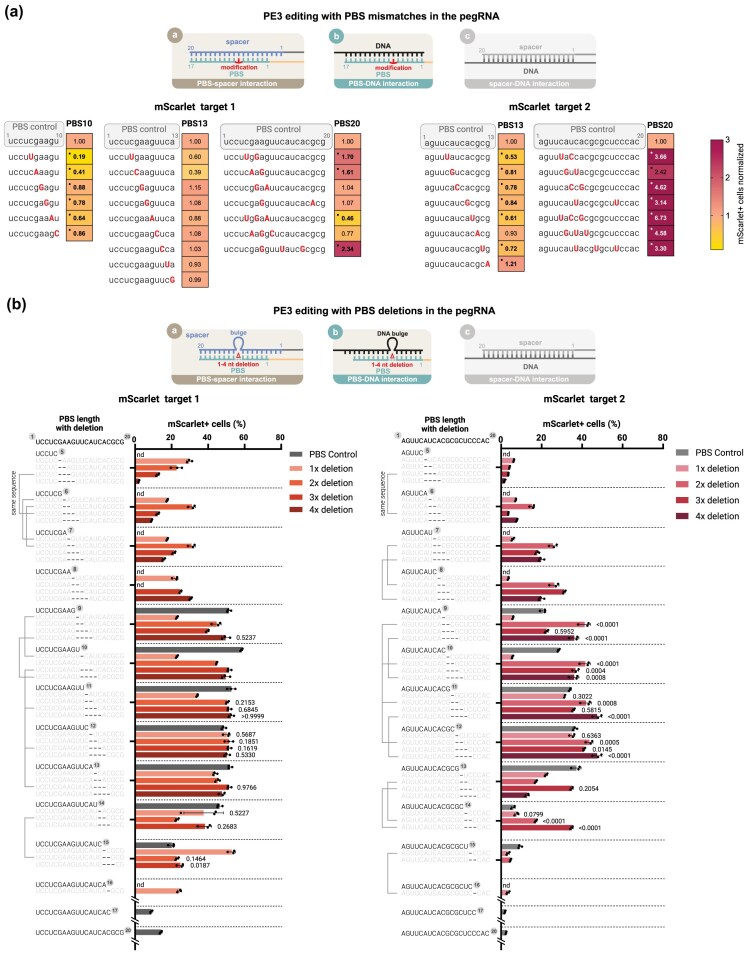
Mismatches in a 20 nt PBS may increase the efficiency of prime editing, while small deletions can reach the efficiency of the optimal pegRNA. Experiments were performed using PE3 on the plasmid-based PEAR system in HEK293T cells. The three small schematic figures above the heat maps and bar charts show the interacting sequences that may be affected by the modifications (those not affected are coloured grey). (**a**) Introduction of mismatches into the PBS sequence for the two *mScarlet* targets. In the case of PBS20, multiple mismatches were used simultaneously. PBS sequences are shown on the vertical axis in a 5′-3′ orientation, with mismatches in capital letters and highlighted in red. The PE efficiency values shown in the heatmaps were normalized to their corresponding no-mismatch-containing pegRNA control values. A significant deviation from the control is indicated by a dot in the upper left corner. (**b**) Effect of 1–4 nt deletions in the long (20 nt) PBS sequence, for the two *mScarlet* targets. The PBS sequences are shown in a 5′-3′ orientation on the vertical axis. Sequences in black indicate the PBS segments that remained intact upstream of the position of the deletion(s) (the number following the sequence represents its length); they also show the PBS of the pegRNAs used as controls. The thin black lines on the left side of the panels connect the cases where different deletions resulted in the same exact sequence. The bars show the mean and mean ± SD of *n* = 3 samples. The *P* values are shown above the columns, indicating deviations from the no-deletion-containing control. Differences between the control and the samples containing a mismatching PBS (a) or between pegRNAs with control PBS length and samples with PBS20 containing deletion(s) (b) were tested for significance using one-way ANOVA or Kruskal–Wallis test. For all measured data, *P* values, and Supplementary Figs S3 and S4, and Supplementary Data. n.d.: no data.

We examined whether, as a result of the aforementioned three effects, we would observe an increase in PE3 efficiency using both shorter (10 and 13 nt) and longer (20 nt) PBSs and the three formerly selected targets. For the two *mScarlet* targets, with the shorter PBSs (PBS10 and PBS13), mismatches rarely increased PE efficiency significantly (in 1 out of the 23 mismatching pegRNAs tested), most of them significantly reduced it (Fig. 2a, Supplementary Fig. S4), and with longer PBSs (PBS20), mismatches increased editing by up to 6.7-fold, with only 4 of the 14 mismatched pegRNAs failing to reach at least a 1.5-fold improvement. The negative effects appeared to be more pronounced with shorter PBSs, in which the inhibitory complementary interaction is inherently weaker. In contrast, with longer PBSs, the inhibitory effect is stronger and the RNA:DNA heteroduplex remains more stable even in the presence of mismatches. Interestingly, in the case of the GFP target with the longer PBS, mismatches at the same positions abolished editing efficiency (Supplementary Fig. S3b).

These results strongly support the idea that the intramolecular complementarity of the PBS to the spacer can have an inhibitory effect on the efficiency of prime editing, reinforcing conclusions drawn from previous studies using different experimental approaches [16, 27].

### Deletions in the PBS

Next, we examined how creating deletions instead of mismatches in the long PBSs (PBS20) would alter the effect of spacer-PBS complementarity relative to the DNA:PBS heteroduplex. Consecutive deletions ranging from 1 to 4 nucleotides in length were introduced starting from position 6 to 15 of the PBS sequence (Supplementary Fig. S5, S6a). For comparison, we also present results from experiments using PBSs with lengths that matched the intact PBS segments upstream of the introduced deletions. Similarly to mismatches, deletions also increased editing efficiency at several positions in comparison to the no-deletion-containing long PBS pegRNA, either approximating or even exceeding the efficiency achieved with the optimal PBS (Fig. 2b, Supplementary Fig. S6b). Deletions starting at the 13th position yielded some of the highest efficiencies for both targets, and therefore, we focused on deletions at this position for further investigation.

**Figure 3.**
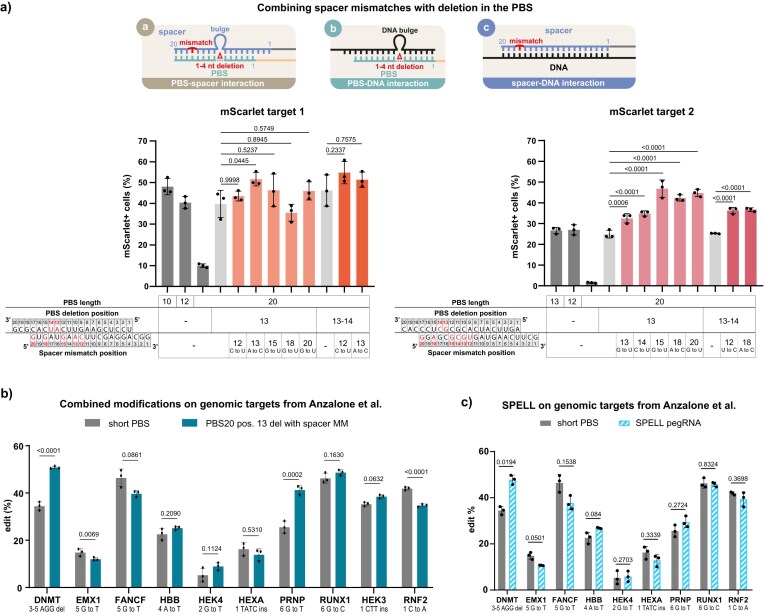
Combination of spacer mismatches and PBS20 deletions could lead to further enhancement of prime editing. (**a**) The effect of combining 1 or 2 nt deletions in PBS20 with spacer mismatches on PE3 activity using the plasmid-based PEAR system in HEK293T cells. The altered PBS and spacer positions are highlighted in red. The *P* values are shown above the columns, indicating deviations from the control containing only the deletion. The three small schematic figures at the top show the interacting sequences that may be affected by the modifications. (**b, c**) Effect of the combination of spacer mismatches (transitions or transversions) and a single nucleotide PBS20 deletion at position 13 on 10 endogenous targets in HEK293T cells. Here, we show only the results of PE3 editing for pegRNAs derived from Anzalone *et al.* with a shorter PBS (*DNMT*: PBS13, *EMX1*: PBS15, *FANCF*: PBS14, *HBB*: PBS8, *HEXA*: PBS12, *PRNP*: PBS12, *RUNX1*: PBS17, *HEK3*: PBS13, *RNF2*: PBS15) and for the best-performing combinations (b) or the PBS-deletion-only SPELL pegRNAs (c). Further details are provided in Supplementary Fig. S7b. The *P* values are shown above the columns, indicating deviations from the short PBS control. (a, b, c) The bars show the mean and mean ± SD of *n* = 3 samples. The values were determined by using next-generation sequencing. Differences between the samples were tested for significance using one-way ANOVA or Kruskal–Wallis test. For all measured data and *P* values, see Supplementary Fig. S7 and Supplementary Data..

We also investigated whether the editing efficiency-enhancing effects of spacer mismatches and PBS deletions are cumulative when combined (Fig. 3a). Our results showed that all tested mismatches significantly increased the editing efficiency of the deletion-only pegRNAs for *mScarlet* target 2, while none of the added mismatches caused a significant decrease in prime editing activity compared to the deletion-only pegRNAs for the two examined targets. The same outcomes were observed when these pegRNAs were compared to those of optimal PBS lengths (Supplementary Fig. S7a). Therefore, based on the PEAR experiments, the most effective strategy seems to be introducing a mismatch mutation into the spacer and a deletion mutation into the PBS.

### Decreasing the spacer-PBS complementarity enhances editing efficiency for genomic targets

We also explored the potential to enhance editing by disrupting the intramolecular self-complementary interaction between the spacer and PBS on genomic targets in HEK293T and HuES cells.

We repeated editing experiments on 10 genomic targets previously characterized in the literature [1], using both the pegRNAs employed in the original study and newly designed pegRNAs, modified to reduce the spacer-PBS complementarity in HEK293T cells. These new pegRNAs contained a deletion at position 13 in a 20-nucleotide-long PBS and an additional transition or transversion mismatch in the spacer at either position 13, 15, or 18 (Supplementary Fig. S7b).

Two out of the 10 edits showed significantly higher editing with pegRNAs harbouring both modified spacer and PBS than the pegRNA with the literature-derived optimal PBSs (Fig. 3b). This, together with the PEAR experiments in Supplementary Fig. S7a, indicates that by combining spacer and PBS modifications, it is possible to achieve higher editing efficiency than with the traditional pegRNAs with optimal PBS. Although it apparently requires further extensive optimization.

Comparing these pegRNAs modified in both the spacer and PBS regions to the corresponding deletion-only pegRNAs, however, showed that the increased efficiency of the altered pegRNAs seen in Fig. 3b did not necessarily require the additional mismatches. Only two pegRNAs had a significant increase in editing efficiency (*PRNP* target) compared to the corresponding pegRNAs without the additional mismatch (Supplementary Fig. S7b).

The pegRNAs with only the PBS deletion achieved editing efficiencies similar to the literature-tested pegRNAs in all cases (Fig. 3c). This suggests that using a 20-nucleotide-long PBS with a deletion may offer an efficient alternative, approximating the performance of the optimal PBS, and it also reduces the burden of optimizing the combination of modifications. We termed this approach SPELL (Streamlined Prime Editing with fixed-Length PBS Leverage).

**Figure 4.**
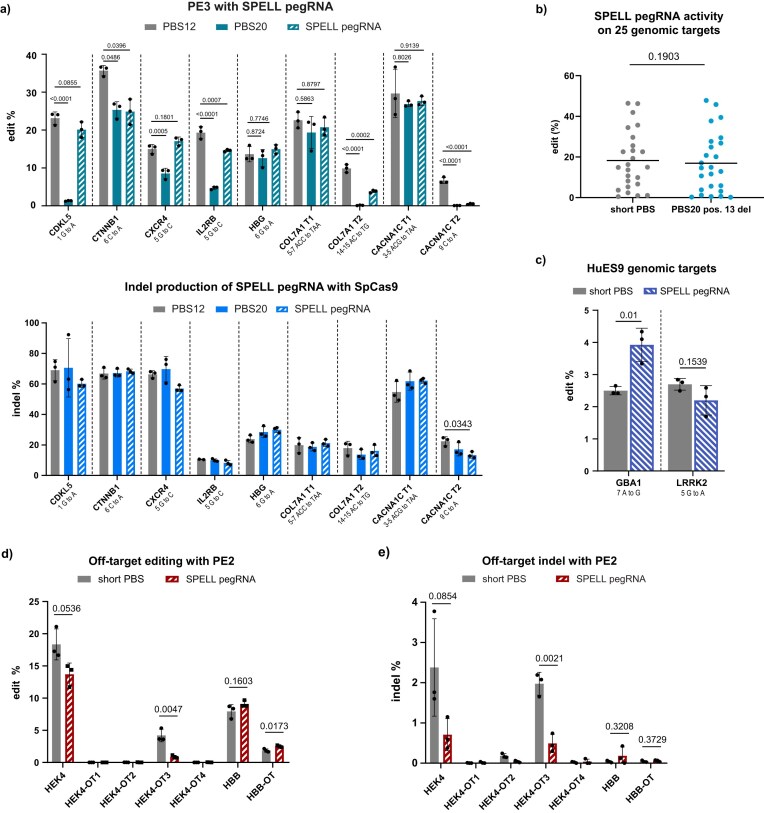
PegRNAs with PBS20 and a single deletion can achieve the efficiency of pegRNAs with optimal PBSs. (**a**) Results of PE3 editing (upper panel) and SpCas9 cleavage (lower panel) at nine genomic targets in HEK293T cells, using pegRNAs with two different PBS lengths and SPELL pegRNA as indicated in the figure. The *P* values are shown above the columns, indicating deviations from the PBS12 control. (**b**) Summarized short PBS and SPELL pegRNA PE3 editing results on genomic targets. Data are derived from experiments shown in Supplementary Figs S7b and S8a. (**c**) PE3 editing at two genomic targets using pegRNAs with short PBS (PBS13) or deletion-only SPELL pegRNAs in a hPSC line. (**d, e**) Off-target editing and indel with PE2 at previously characterized off-target sites, *HEK4* [1] and *HBB* [37], using pegRNA with short PBSs (*HEK4*: PBS8, *HBB*: PBS13) and SPELL pegRNA. (a, c, d, and e) The bars show the mean and mean ± SD of *n* = 3 samples. The values were determined by using next-generation sequencing. Differences between the samples were tested for significance using an unpaired two-tailed t-test (a, d, e), a paired two-tailed t-test (b), one-way ANOVA, or the Kruskal–Wallis test (c). For all measured data and *P* values, see Supplementary Figs S7b and S8 and Supplementary Data.

To further test SPELL, we compared the efficiency of seven previously employed pegRNAs [34] targeting the *CYP1A1, CYP1A2, CYP2B6, RYR2*, and *KRT12* genes to new pegRNAs designed to achieve the same edit but with a 20-nucleotide-long PBS containing a deletion at position 13. In 4 out of 7 cases, the pegRNAs with the long, non-optimized PBS performed similarly to their original counterparts, while three showed significantly lower editing efficiency (Supplementary Fig. S8a). We further examined an additional set of nine pegRNAs, five of which (*CDKL1, CTNNB1, CXCR4, IL2RB*, and *HBG*) had been characterized previously [14, 35], and compared their activity to that of the corresponding SPELL pegRNAs. In this set, the majority of SPELL pegRNAs exhibited editing efficiencies comparable to pegRNAs with the short PBS (Fig. 4a).

Altogether, across all 25 pegRNAs analyzed in HEK293T cells, no significant difference in overall activity was observed between pegRNAs with a short PBS and those with a 20-nt PBS containing the deletion at position 13 (SPELL pegRNAs) (Fig. 4b).

Additionally, we tested two targets in the *LRRK2* and *GBA1* [36] genes in HuES cells. For the *GBA1* target, the pegRNAs with the long, non-optimized, single-deletion-containing PBS achieved a 1.5-fold improvement in editing efficiency, compared to literature-derived PBS, while in the case of the *LRRK2* gene, there was no significant difference (Fig. 4c).

Examining the indel background of the pegRNAs analyzed above revealed no indication that SPELL pegRNAs would exhibit increased indel formation (Supplementary Figs S7b and S8b–d). Furthermore, neither off-target editing (Fig. 4d) nor off-target indel formation (Fig. 4e) appeared elevated when using SPELL pegRNAs, as compared to the two pegRNAs known from the literature [1, 37].

### RNP delivery of the pegRNAs

We also tested the SPELL approach using RNP delivery. An additional consideration arose from the study of Ponnienselvan *et al.*, as they reported that when purified protein or mRNA-encoded prime editor was complexed with synthetic, end-protected pegRNAs, the optimal PBS length differed from that observed with plasmid-delivered pegRNAs, which lack end protection, for both targets they examined. However, importantly, when describing RNP or RNA delivery, their work consistently used synthetic, end-protected pegRNAs; therefore, the effects of delivery method and end protection were not examined independently.

To investigate how the delivery method affects editing efficiency using various pegRNAs, we performed RNP-based editing experiments on five targets using 3′-unprotected *in vitro*–transcribed pegRNAs with short PBSs (7 and 8 nt), pegRNAs with PBS12, and SPELL pegRNAs. In two of the five cases, the tested pegRNAs showed similar performance patterns between plasmid and RNP delivery (Supplementary Fig. S8e; *CDKL5* and *COL7A1 T2*). For one target, however, the 8 nt PBS pegRNA yielded higher editing efficiency than PBS12 when delivered as an RNP (Supplementary Fig. S8e; *CACNA1C T2*). In contrast, for two targets, RNP delivery reduced the activity of pegRNAs with short, 7 or 8-nucleotide PBSs (Supplementary Fig. S8f).

These observations indicate that, in some cases, the delivery method may influence which PBS length performs best for a given target. A more comprehensive investigation, particularly regarding the performance of SPELL pegRNAs in the context of RNP delivery, will require further systematic study.

### Deletions may act on multiple routes

To investigate how the weakened spacer:PBS annealing and the PBS region downstream of the deletion site contribute to the activity of SPELL pegRNAs, we performed several additional experiments.

In Fig. 2b, we directly compared pegRNAs carrying the non-altered, intact PBS region to their corresponding PBS-deletion pegRNAs. In this sense, a pegRNA with intact PBS corresponds to a SPELL pegRNA where the PBS segment upstream of the deletion is the same; for example, a deletion at position 13 in a PBS20 construct corresponds to an intact 12 nt PBS. In 6 out of the 13 comparisons, the PBS-deletion pegRNAs yielded significantly higher editing efficiencies than their intact-PBS counterparts. This indicates that, in several cases, the 3′ end region of the PBS (i.e. the nucleotides downstream of the SPELL deletion site) contributes to activity. Although the most intuitive interpretation is that this increase in activity results from the annealing of the PBS end to the non-target strand, the mechanism underlying this contribution is not clear.

We further examined the role of the 3′ end region of the PBS by scrambling this segment in three pegRNAs (Supplementary Fig. S8g). For one pegRNA, scrambling the PBS end reduced editing efficiency below that of the pegRNA with the intact PBS. For the second pegRNA, scrambling increased editing efficiency even further, whereas for the third one, scrambling had no significant effect. These results indicate that the PBS end region in SPELL pegRNAs may either enhance or reduce activity through effects that are independent of PBS:DNA annealing (e.g. pegRNA stability or protein-RNA interactions). It is also likely that the PBS end region mediates multiple effects simultaneously, with the relative contribution of each varying across pegRNAs.

We also investigated whether the deletion at position 13 influences SpCas9 nuclease activity (Fig. 4a). Interestingly, we did not observe reduced nuclease activity with the 20 nt PBS compared to shorter PBSs, as would be expected from increased PBS:spacer inhibition, nor did the SPELL deletion increase nuclease activity relative to PBS20, as would be expected from reduced PBS:spacer inhibition, for any of the pegRNAs tested.

At first sight, this may appear unexpected, given that in 5 out of the 9 targets PBS20 pegRNAs showed reduced prime editing activity, and in 4 of these cases the SPELL deletion increased editing efficiency (Fig. 4a). Our interpretation is that the increased inhibition of prime editing by stronger spacer:PBS interactions does not necessarily manifest at the level of SpCas9 nicking. This conclusion is reminiscent of our earlier findings [38], where RTT-scaffold interactions strongly inhibited prime editing efficiency while having no observable effect on nuclease activity.

These observations suggest that, for certain targets, likely including several examined here, the inhibitory effect of the increasing PBS length, and thereby the strengthening of spacer:PBS interactions, does not further impair SpCas9 nicking. Instead, the inhibition appears to act at a subsequent step of the prime editing process, most plausibly by interfering with efficient RT priming.

In conclusion, our results strongly support the presence of an inhibitory effect exerted by complementary spacer-PBS interactions in prime editing. We demonstrate that reducing these interactions can substantially improve editing efficiency, and that such modified pegRNAs can outperform pegRNAs containing the otherwise optimal PBS. However, achieving this improvement requires extensive optimization.

Our findings partially overlap with those of Fei *et al.* [39], who primarily focused on spacer:protospacer interactions. In their study, the edits introduced by prime editing typically altered the target sequence (positions 1–3 and 5–6), and mismatches added to the spacer further reduced re-nicking of the target site. They also speculated that mismatches at certain positions may affect the stability of the spacer:protospacer duplex, and that duplex flexibility, as predicted by AlphaFold, may correlate with prime editing outcomes. Consistent with their observations, we also found that spacer-mismatched pegRNAs can reduce indel formation at several targets, although indels may increase at others (Supplementary Fig. S7b).

However, Fei *et al.* did not consider the effects of PBS length or PBS melting temperature—factors that determine the effective position and the impact of the introduced mismatches—nor did they explicitly report on these parameters. In contrast to their emphasis on spacer–protospacer interactions, our work focuses on inhibition arising from spacer:PBS complementarity. Our results also caution against drawing firm conclusions from a limited number of targets or pegRNAs and demonstrate that altering the sequence of either the spacer or the PBS can have multiple consequences beyond simply weakening intramolecular spacer:PBS interactions.

SpCas9 is generally tolerant of mismatches, particularly those located at PAM-distal positions. Thus, it was somewhat unexpected that the *GFP* target did not tolerate spacer mismatches without a reduction in cleavage activity. The sgRNAs and pegRNAs targeting this locus contained an appended 5′ G nucleotide to satisfy the U6 promoter requirement [40], resulting in a 21-nt spacer. Fu *et al.* demonstrated that a 5′ GG extension increases nuclease fidelity by weakening off-target cleavage [40], and we subsequently showed that appending a single 5′ G can similarly enhance fidelity [41]. The *GFP* target may therefore display increased sensitivity to mismatches because of its 21-nt spacer length. Although the effect of a 5′ G extension can depend on the intrinsic cleavability ranking of the target [42], pegRNAs with 21-nt spacers may be less amenable to our approach.

We also observed that pegRNAs with 17-nt PBSs exhibited reduced nuclease activity (Fig. 1c). Surprisingly, however, altering the strength of the spacer:PBS interaction—despite its clear influence on prime editing efficiency—did not seem to affect cleavage activity among pegRNAs sharing the same spacer sequence (Fig. 4a). This suggests that spacer:PBS complementarity also influences steps other than the initial SpCas9 nicking.

Finally, our study demonstrates that high editing efficiency can be achieved for many targets without extensive optimization by using SPELL—a simplified strategy based on a single-nucleotide PBS deletion.

## Supplementary Material

gkag292_Supplemental_Files

## Data Availability

All data is available in the paper’s Supplementary data files. The deep sequencing data have been submitted to the NCBI Sequence Read Archive and are available via accession number: PRJNA1196934.
